# Development of a Novel Severe Triple Allergen Asthma Model in Mice Which Is Resistant to Dexamethasone and Partially Resistant to TLR7 and TLR9 Agonist Treatment

**DOI:** 10.1371/journal.pone.0091223

**Published:** 2014-03-11

**Authors:** Matthias J. Duechs, Cornelia Tilp, Christopher Tomsic, Florian Gantner, Klaus J. Erb

**Affiliations:** Respiratory Diseases Research, Boehringer Ingelheim Pharma GmbH & Co. KG, Biberach a.d. Riss, Germany; French National Centre for Scientific Research, France

## Abstract

Severe asthma is characterised by persistent inflammation, hyperreactivity and remodeling of the airways. No efficient treatment is available, this is particularly the case for steroid resistant phenotypes. Our aim therefore was to develop a preclinical model showing characteristics of severe human asthma including steroid insensitivity. Mice were first sensitized with ovalbumin, extracts of cockroach or house dust mite followed by a challenge period of seven weeks. Further to this, an additional group of mice was sensitized with all three allergens and then challenged with allergen alternating weekly between allergens. All three allergens applied separately to the mice induced comparably strong Th2-type airway inflammation, airway hyperreactivity and airway remodeling, which was characterised by fibrosis and increased smooth muscle thickness. In contrast, application of all three allergens together resulted in a greater Th2 response and increased airway hyperreactivity and a stronger albeit not significant remodeling phenotype compared to using HDM or CRA. In this triple allergen model dexamethasone application, during the last 4 weeks of challenge, showed no suppressive effects on any of these parameters in this model. In contrast, both TLR7 agonist resiquimod and TLR9 agonist CpG-ODN reduced allergen-specific IgE, eosinophils, and collagen I in the lungs. The TLR9 agonist also reduced IL-4 and IL-5 whilst increasing IFN-γ and strongly IL-10 levels in the lungs, effects not seen with the TLR7 agonist. However, neither TLR agonist had any effect on airway hyperreactivity and airway smooth muscle mass. In conclusion we have developed a severe asthma model, which is steroid resistant and only partially sensitive to TLR7 and TLR9 agonist treatment. This model may be particular useful to test new potential therapeutics aiming at treating steroid resistant asthma in humans and investigating the underlying mechanisms responsible for steroid insensitivity.

## Introduction

Severe asthma is characterised by persistent inflammation of the airways, airway hyperreactivity and pronounced airway remodeling, involving increased fibrosis and smooth muscle thickening, mucus overproduction and sub epithelial thickening. Patients with this asthma phenotype often exhibit a steroid resistance and are prone to acute exacerbations, leading to the highest mortality rates. These patients account for approximately 30% of the overall healthcare costs caused by asthma [1]. For this reason there has been a strong emphasis on trying to develop novel treatment options specifically for severe asthma. However, thus far most therapies have no or only marginal therapeutic effects. Aside from the anti-IgE therapy for asthmatics with high IgE levels no new drug has been approved for asthma in the last 10 years. Phase 2 studies suggest that an anti-IL-13 therapy may help a subpopulation of patients with high periostin levels with severe asthma [2]. Nevertheless, more efficient treatment options for patients suffering from severe asthma and in particular severe, steroid insensitive asthma are required [3].

Murine models display several hallmarks of severe atopic asthma seen in patients. These include Th2 driven pulmonary inflammation with strong lung eosinophilia, elevated levels of Th2 cytokines such as IL-4, IL-5 and IL-13, the production of allergen specific IgE, airway hyperreactivity, and airway remodeling characterized by goblet cell hyperplasia, mucus over production, smooth muscle thickening and sub epithelial fibrosis [4]–[6]. In addition, studies using knockout and transgenic mice have led to the discovery of numerous potential new drug targets to treat severe asthma in patients [7]–[10]. However, many of these new approaches suppressing the allergic responses in mice fail in the clinic, emphasizing the fact that the phenotype induced in mouse models is not identical to the human atopic disease phenotyp [11]. Mimicking severe steroid insensitive asthma in mice is particularly very challenging because the induced phenotype of fibrotic changes and increase in smooth muscle mass is not very pronounced. This leaves a small assay window to measure therapeutic effects. Problems with mouse modeling of a severe asthma phenotype are also compounded by the fact that steroids generally suppress allergic responses in mice very efficiently.

The aim of our study was to identify allergens and an immunization protocol which induces severe asthma in mice, ideally showing a phenotype which is insensitive to steroid treatment. For this purpose BALB/c mice were first sensitized with ovalbumin (OVA), extracts of cockroach (CRA) or house dust mite (HDM) together with alum followed by a challenge period of seven weeks in which mice received the respective allergen via intratracheal administration twice a week. Strong Th2-type airway inflammation, airway hyperreactivity and airway remodeling was observed for all three allergens with no major differences between the allergens. In contrast, when mice were immunized with all three allergens simultaneously and then challenged with allergen (alternating between the three allergens) a greater Th2 response and increased airway hyperreactivity compared to only single allergen was observed. Fibrosis and smooth muscle thickening was enhanced but not significantly compared to HDM or CRA. Furthermore we were interested if this strong phenotype could be supressed by therapeutic intervention. We chose to use dexamethasone, TLR7 (resiquimod; R848) and TLR9 (CpG-ODN) agonists because all three have been previously shown to have potent anti-allergic effects in animal models of asthma [12]–[16]. In addition dexamethasone, a glucocorticoid with potent anti-inflammatory and immunosuppressant properties, is also used for therapy in severe asthma patients. Surprisingly, we found that applying dexamethasone therapeutically in this model had no suppressive effects on the induced asthma phenotype in the lungs. In contrast, both TLR7 and TLR9 agonists reduced some but not all features of the allergic phenotype. Taken together our novel severe asthma model shows a very strong remodeling phenotype which is steroid insensitive and only partially sensitive to TLR7 and TLR9 agonist treatment.

## Methods

### Mice

Female BALB/cAnNCrl mice (Charles River, Sulzfeld, Germany) were maintained under conventional conditions in an isolation facility. At the onset of the experiments, all animals were between 6 and 8 weeks of age.

### Ethics Statement

All experiments were performed according to the guidelines of the local and government authorities for the care and use of experimental animals (Regierungspräsidium Baden-Württemberg, Tübingen, Germany; approval numbers: 09-022; 12-035).

### Allergens

For intraperitoneal sensitization OVA (salt free albumin egg, Serva, Heidelberg, Germany), HDM (mite, house dust, *Dermatophagoides pteronyssinus,* Greer Laboratories, Lenoir, N.C., USA), and CRA (cockroach, german, *Blattella germanica,* Greer Laboratories, Lenoir, N.C., USA) were solubilised in 100 µl phosphate buffered saline and absorbed to 100 µl Al(OH)_3_ (Imject Alum, Rockford, USA). For challenge, allergens were solubilised in 50 µl phosphate buffered saline (PBS, BioWhittaker Europe, Verviers, Belgium) and administered via intratracheal application.

### Treatment protocols

**Figure 1:**
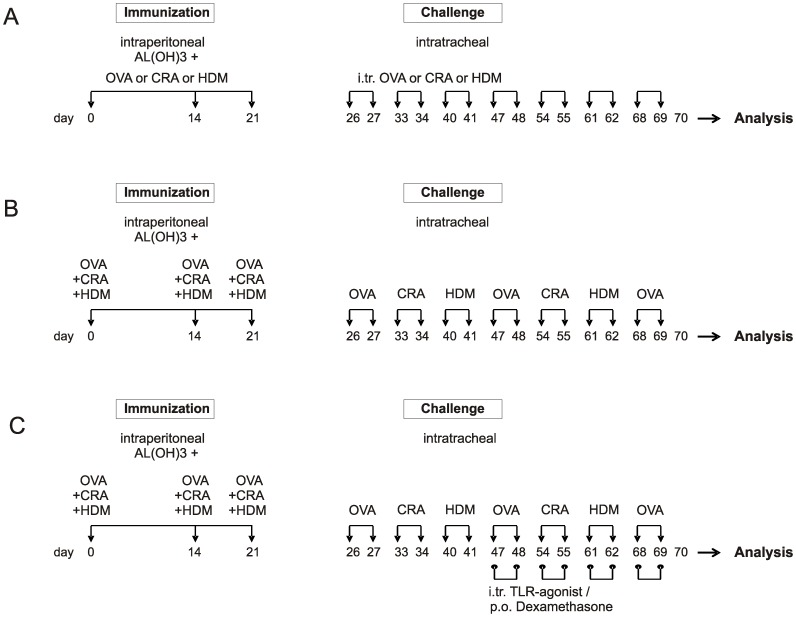
Treatment protocols. Mice were sensitized on day 0, 14 and 21 with ovalbumin (OVA), house dust mite (HDM) or cockroach (CRA) together with alum and challenged intra-tracheally starting on day 26 with the respective allergens twice a week for 7 weeks (A). In the triple allergen combination model (TAC), mice were sensitized with the three extracts plus alum and challenged with one single extract twice a week alternating between the allergens (B). Therapeutic treatment with dexamethasone (per os), R848, or CpG-ODNs (both intratracheal) was started in the 4^th^ week of allergen challenge (C). 1 mg/kg of dexamethasone or TLR agonist was given twice weekly, 1 hour prior to allergen challenge.

Mice were sensitized intraperitoneal on day 0, 14 and 21 with 20 µg OVA, 2.5 µg CRA or 2.5 µg HDM in Alum and challenged intratracheal starting from day 26 with 20 µg of OVA, CRA or HDM twice a week for seven consecutive weeks and sacrificed 24 h after the last challenge (Figure 1A). In the triple allergen combination model mice were sensitized with a mix of 20 µg OVA, 2.5 µg CRA and 2.5 µg HDM extract in Alum intraperitoneally (Figure 1B). Challenge was performed with only one allergen per week, alternating between the three allergens weekly. 1 mg/kg of dexamethasone orally, 1 mg/kg R848, or CpG-ODNs were given intratracheal starting in the 4^th^ week of allergen challenge as described before (12), 1 hour prior to allergen challenge twice weekly (Figure 1C).

### Bronchoalveolar lavage

24 h after the last challenge mice were sacrificed, the trachea cannulated and a bronchoalveolar lavage (BAL) performed by flushing lung and airways twice with 0.8 ml PBS containing 0.6 mM EDTA and 5 mg/ml protease inhibitor (Roche, Mannheim, Germany).

### Quantification of airway eosinophils, macrophages and neutrophils

BAL cells were differentiated after centrifuging 200 μl of BAL fluid on cytospin slides for 1 min at 500 rpm. For differentiation of cells, slides were stained with May-Gruenwald/Giemsa. Cell types were differentiated and counted using light microscopy applying standard morphological criteria. Total counts of cells in BAL fluid were determined using an automated haematology analyzer (Sysmex, Kobe, Japan).

### Immunoglobulin E analysis

Serum levels of allergen specific IgE were measured with ELISA technique on MSD platform using rat anti-mouse IgE (BD Biosciences, Erembodegem, Belgium) and MSD sulfo-tag-labeled goat anti-rat-antibody (Meso Scale Discovery, Gaithersburg, Maryland, USA). In brief, plates were coated with the respective allergen extract (20 µg/ml) and incubated at 4°C over night. Serial dilutions of serum samples were applied and incubated at 4°C over night. Rat anti-mouse IgE (diluted 1∶200) was added and incubated for 45 min at room temperature. Plates were then washed three times before MSD goat anti-rat antibody (diluted 1:500) was added and incubated for 1 h at room temperature. For allergen specific IgE-level determination, plates were analyzed with MSD Sector Imager 6000 (Meso Scale Discovery, Gaithersburg, Maryland, USA). Levels of allergen specific IgE are given in arbitrary units/ml (U/ml), directly correlating to absorbance.

### Preparation of lung tissue homogenate

Lung samples were homogenized with FastPrep-24 (MP Biomedicals, Solon, Ohio, United States of America) using PBS containing 5 mg/ml protease inhibitor (Roche Diagnostics GmbH, Mannheim, Germany).

### Detection of chemokines and cytokines

Chemokines and cytokines were measured in supernatants of homogenized lung tissue using mouse TH1/TH2 9-PlexBase cytokine/chemokine multiplex assay (Meso Scale Discovery, Gaithersburg, Maryland, USA), IL-13 was measured using mouse ELISA Kit (R&D Systems, Minneapolis, USA), and total TGF-β 1 was measured with mouse TGF-ß1 Platinum ELISA (Bender MedSystems GmbH, Vienna, Austria), all according to manufactureŕs instructions.

### Lung histology and immunohistochemistry

After lavage, the left lung of each mouse was fixed in 4% formalin for 24 h and embedded in paraffin wax. All histology samples were prepared from the same position, where the main bronchus enters the lung. Lung sections (2–3 µm) were stained with haematoxylin and eosin (H&E) reagent (Merck, Darmstadt, Germany) for detection of inflammatory infiltrates, with periodic acid-schiff (PAS, Sigma-Aldrich GmbH, Steinheim, Germany) for goblet cells and mucus production. For visualising smooth muscle, α-smooth muscle actin antibody (Clone 1A4, Dako, Glostrup, Denmark) was used. For visualizing collagen, a picro-sirius red staining with picric acid (Sigma-Aldrich, Steinheim, Germany) was performed. Cross sections showing the main bronchus were used for quantification of pathological changes. For quantification of smooth muscle thickening and mucus production automatic analyses were performed using Halcon software (MVTec Software GmbH, Munich, Germany). Airway smooth muscle thickness was quantified by assessing diameter of the muscle layer surrounding the main bronchus. In brief, an inner line at the border of airway space (lumen) and epithelium was drawn. In 300 µm distances to the inner circle a second circle surrounding the main bronchus area was drawn. Following this, vertical auxiliary lines in intervals of 2 µm were drawn from the inner to the second circle. For quantification, the length of stained auxiliary line was measured. Following the mean ± standard error of means (SEM) of all measured lengths were calculated. For quantification of mucus, epithelial layers of the PAS stained lung slices were defined by delimiting the area from lumen to sub epithelial tissue. Following this step, areas of epithelial layers and total red stained areas were quantified. The ratio of mucus and bronchus area was calculated. Mucus plugging was scored manually by semi-quantitative analysis (double-blinded). The left lung of a mouse was considered plugged when more than 75% of the main bronchus was filled with mucus. Levels of fibrosis are expressed as collagen to void area in %, a value calculated as follows: sub epithelial collagen area ( µm^2^)/void area ( µm^2^) x 100. The void area is the area of the main bronchus free airway lumen.

### Airway hyperreactivity measurements

Airway hyperreactivity (AHR) to methacholine was assessed using whole-body-plethysmography (Buxco, Wimington, NC, USA) as described previously [17]. AHR was further assessed by invasive measurement of resistance and compliance using FinePointe R/C systems (Buxco, Wimington, NC, USA). Animals were anesthetized by intraperitoneal injection and after intubation an oesophagus catheter was introduced to measure pleural pressure. For measurement of resistance and compliance mice were exposed to methacholine in increasing concentrations from 0.625 to 12.5 mg/mL. Resistance was measured in [cm H2O/ml/sec] and compliance was measured in [ml/cm H_2_O].

### Collagen analysis

Collagen levels were assessed in supernatant of homogenized lung tissue using ELISA technology based mouse type I and III collagen detection kits (Chondrex, Redmond, USA and Cusabio, China) according to manufacturer`s instructions.

### Statistics

Data analyses were performed using the Prism 4 software package (GraphPad Software, San Diego, CA). If not stated otherwise, One-way analysis of variance (ANOVA) with the Dunnett’s-post test was used to determine statistical difference to reference group (negative control or untreated). A P value 0.05 was considered statistically significant.

## Results

### Combination of three allergens causes the strongest Th2 type inflammation in the airways compared to single allergen use

**Figure 2:**
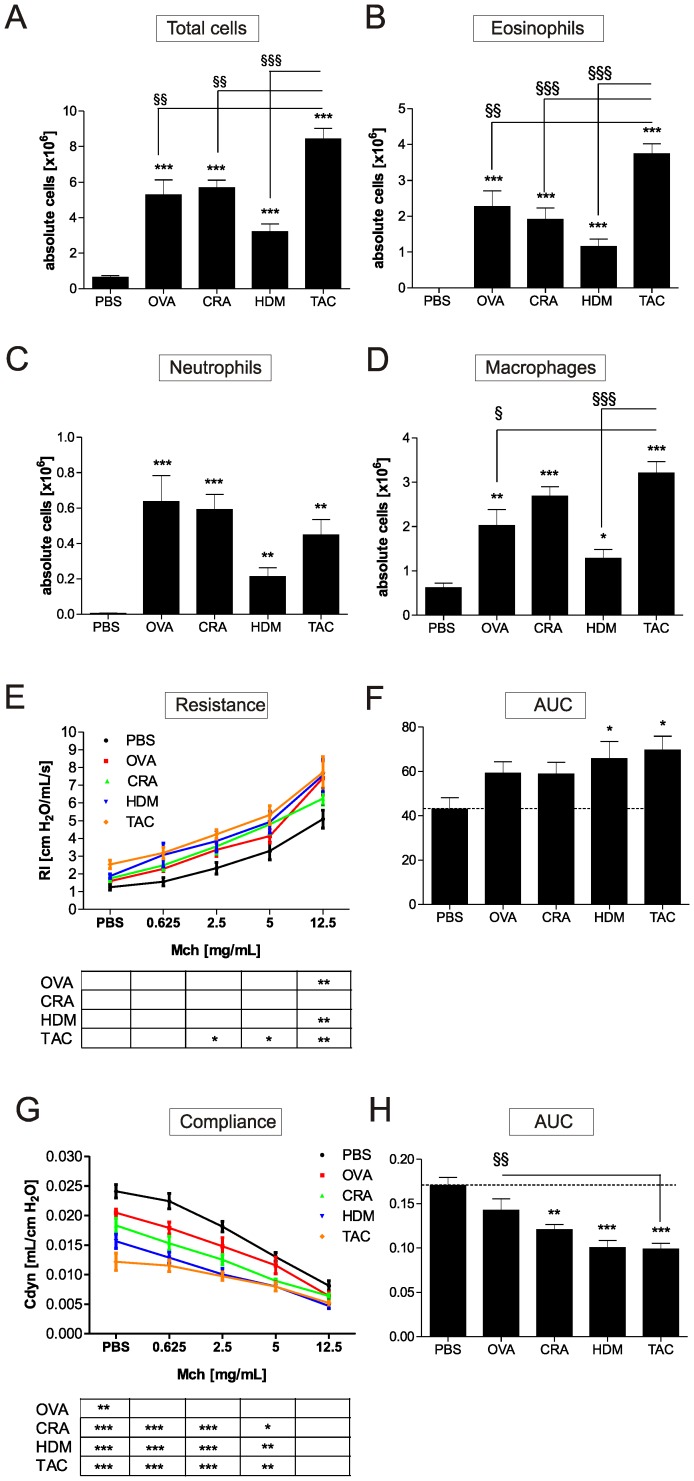
The triple allergen combination model induces the strongest AHR and influx of eosinophils into the lung. 24 h after the last allergen challenge mice were sacrificed and absolute numbers of total cells (A), eosinophils (B), neutrophils (C) and macrophages (D) were measured in whole lung lavage. Lung function was assessed 24 h after final challenge on day 70. Invasive measurement of AHR was used to assess resistance (E) and compliance (G), which was further evaluated using area under the curve (AUC) (F, H). Results represent mean ± SEM from 8–12 mice/group. *P<0.05; **P<0.01; ***P<0.001 in comparison to the PBS control mice, and ^§^ *P*<0.05; ^§§^ *P*<0.01, ^§§§^ *P*<0.001 in comparison to the triple allergen combination (TAC) group.

In order to develop a preclinical model of severe human asthma BALB/c mice were treated either with OVA, extracts of CRA, HDM (Figure 1A), or a combination of all three (Figure 1B). We chose this immunization protocol because we have previously found that when using this approach and OVA a severe asthma phenotype was induced [18]. The single allergens and the combination were compared with respect to their ability to induce pulmonary inflammation and remodeling. In the models presented here intraperitoneal sensitization was conducted together with alum. OVA, CRA, and HDM, individually applied, induced significant influx of eosinophils, neutrophils and macrophages into the lung and increased the amount of IL-4 and IL-5 detected in lung homogenates (Figure 2 and Table 1). The combination of all three allergens resulted in the highest counts of total cells, eosinophils and macrophages in the lung (Figure 2). Compared to single allergen treatment the combination of the allergens also resulted in 3-fold higher levels of IL-4 and IL-5 in the lungs and lead to a significant increase in IL-13, IFN-γ, IL-12 and IL-2 (Table 1).

**Table 1:** Levels of cytokines in the lungs of mice subjected to the different sensitization and challenge protocols.

	PBS	OVA	CRA	HDM	TAC
**IL-4** [pg/ml]	3.12±1.04	57.43±12.54 (**,§§§)	55.20±8.51 (***,§§§)	64.15±6.99 (***,§§§)	239.1±26.54 (***)
**IL-5** [pg/ml]	1.02±1.02	130.20±33.83 (**,§§)	74.57±10.57 (***,§§§)	81.15±11.5 (***,§§§)	345.50±61.15 (***)
**IL-10** [pg/ml]	25.08±3.56	158.04±54.66	131.84±23.13	191.64±27.35	141.41±23.94
**IL-13** [pg/ml]	3412±475.9	5198±699.5	4951±1451	6125±793.7	8310±1245 (***)
**TGF-β1** [ng/ml]	134.4±25.55	125.3±16.23	140.9±14.33	229.6±65.15 (**)	205.2±46.52 (*)
**IFN-γ** [pg/ml]	2.41±0.52	0.65±0.3 (§§)	5.44±2.1 (§)	5.56±1.67(§)	21.06±6.06 (**)
**IL-2** [pg/ml]	10.73±1.95	4.34±0.85 (§§)	4.54±1.0 (§§)	6.04±1.62 (§§)	44.51±14.12 (**)
**IL-12** [pg/ml]	836.0±290.4	962.8±106.5 (§)	1284±125.7	1453±139.6	1649±167.0 (**)
**IL-17** [pg/ml]	501.2±70.49	1458±110.9 (***)	1303±117.8 (***)	1038±82.58 (***)	858.7±78.4 (*)
**IL-1ß** [pg/ml]	544.17±154.49	1896.23±697.92	1590.43±347.75	1410.88±326.86	933.67±153.81

Values are expressed as mean ± SEM from 8–14 mice per group. *P <0.05, **P <0.01, ***P<0.001 in comparison to the PBS control and §< 0.05, §§< 0.01, and §§§<0.001 compared to the triple allergen combination (TAC) group.

### Triple allergen combination induces a strong hyperreactivity of the lung

Compared to using the single allergens mice treated with the combined allergens showed highest baseline pulmonary resistance and lowest baseline compliance. After methacholine challenge these mice also showed the highest increase in resistance (Figure 2E). Resistance was also significantly elevated in OVA and HDM treated mice at the highest concentration of methacholine. Compliance was significantly decreased in all groups and was lowest in the HDM and combination treated mice (Figure 2G). The overall strongest resistance and lowest compliance, using area under the curve, was measured in the HDM and combination treated mice (Figure 2F and H).

### Combination of three allergens leads to strong airway remodeling

**Figure 3:**
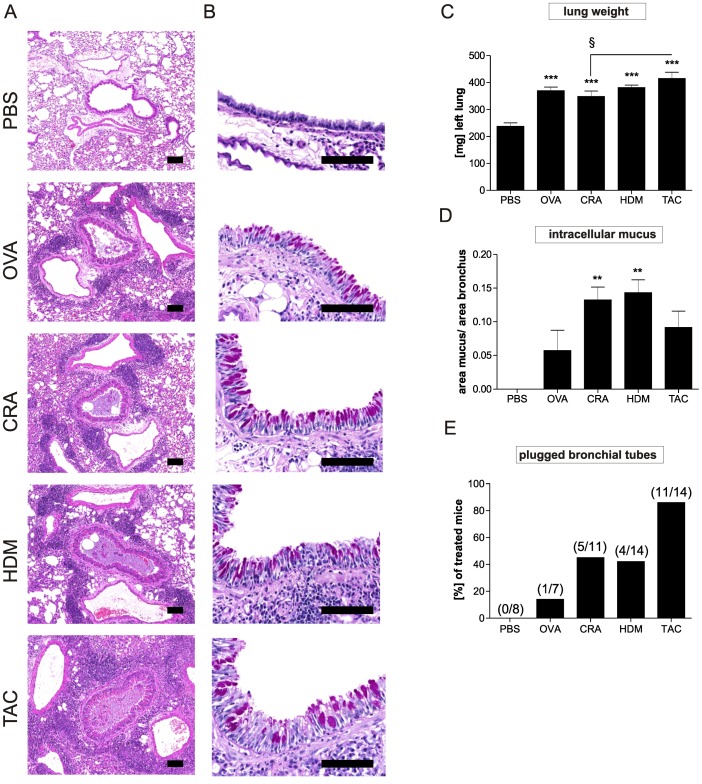
Inflammation and mucus production in the lung of the mice subjected to the different sensitization and challenge protocols. Sections of the lungs were stained with haematoxilin/eosin (H&E) (A) and periodic acid-schiff (PAS) (B). Shown are representative sections of 8–12 mice/group. Scale bar  =  100 µm. Wet weight of left lung is shown (C) and mucus production (D) was assessed by automated analysis of the main bronchus epithelium. Results represent mean ± SEM from 8–12 mice/group. **P<0.01; ***P<0.001 in comparison to the PBS control mice and ^§^ *P*<0.05 in comparison to the triple allergen combination (TAC) group. Mucus plugging (E) was scored manually analyzing whole lung cross sections from 7–12 mice/group as described in methods.

**Figure 4:**
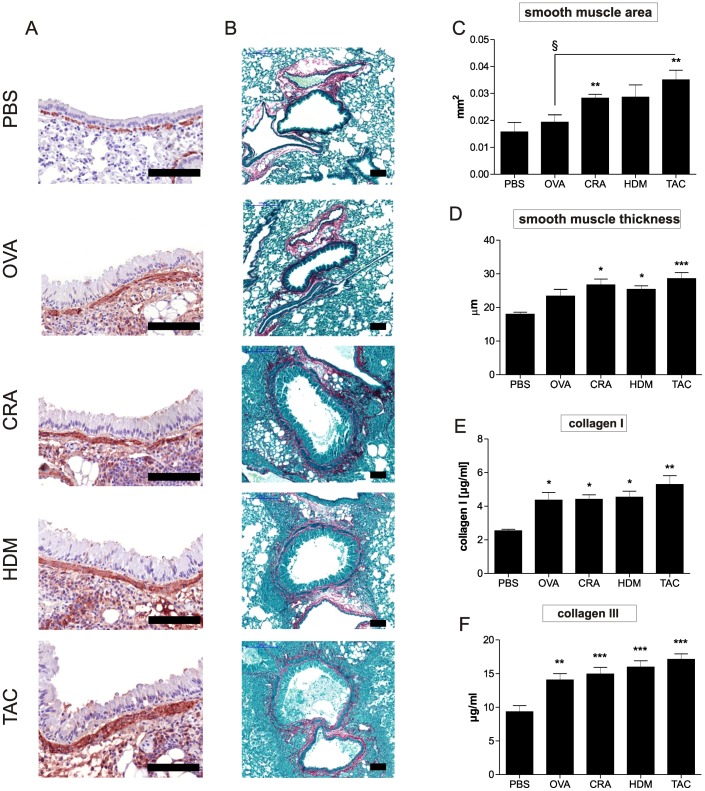
Smooth muscle thickening and lung fibrosis is strongest in the mice treated with all three allergens. Smooth muscle increase was detected by α-smooth muscle actin staining (SMA) (A). Sirius red staining was used to detect sub epithelial collagen deposition (B). Shown are representative sections of 8–12 mice/group. Scale bar  =  100 µm. Smooth muscle area (C) and smooth muscle thickness (D) were assessed by automated analysis of α-smooth muscle actin stained lungs. Fibrosis was quantified by ELISA measurement of collagen I (E) and collagen III (F) in lung homogenates. Results represent mean ± SEM from 8–14 mice/group. *P<0.05; **P<0.01; ***P<0.001 in comparison to the PBS control mice and ^§^ *P*<0.05in comparison to the triple allergen combination (TAC) group.

The wet weight of the left lung showed significant increase in all treated mice with the highest increase detected in the group treated with all three allergens. The highest cellular influx into lung tissue was also observed in the combination model (Figure 3A). In addition, all groups showed an increase in mucus production. The highest amounts of intracellular mucus were detected in HDM and CRA treated animals, whereas OVA and the combination treated animals exhibited the lowest amount of intracellular mucus (Figure 3D). However, the strongest mucus plugging was observed in the combination model (Figure 3E) which induced plugging in 11 of 14 mice (PBS in 0 of 8, OVA in 1 of 7, CRA in 5 of 11 and HDM in 6 of 14), suggesting that here the reduced amount of intracellular mucus detected in the goblet cells may be due to the secretion of the mucus. Periodic acid Schiff staining also showed increased mucus production in the epithelium of the main bronchus as well as a distinct hyperplasia in all groups of mice (Figure 3B). Compared to sham treated mice all models induced thickening of smooth muscle layers albeit to different degrees (Figure 4A, C, D). CRA and combination treatment led to the strongest increase in smooth muscle mass. Compared to untreated mice, all treatments induced significant increase in collagen I and collagen III levels. The highest increase of collagen I and collagen III in lung tissue was again measured in the combination model (Figure 4E and F). TGF-ß1 was only significantly increased in the HDM and combination model (Table 1).

### The asthmatic phenotype induced by the combination of allergens is steroid insensitive and only partially sensitive to TLR7 and TLR9 agonist treatment

**Figure 5:**
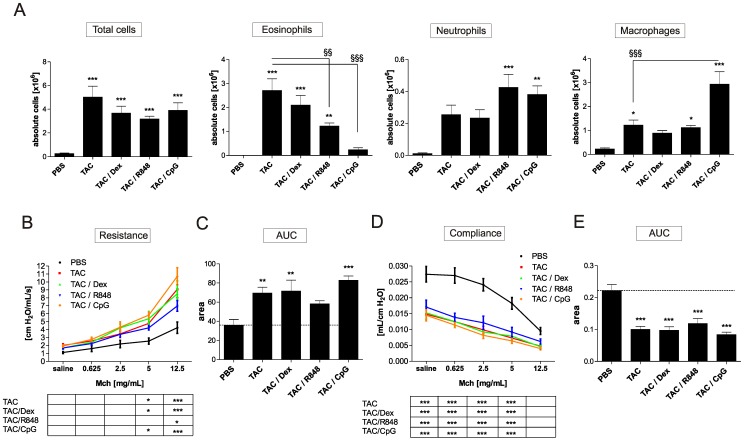
Effects of dexamethasone, TLR7 or TLR9 agonists on cellular influx and airway hyperreactivity in mice subjected to the triple allergen challenge model. Therapeutic treatment with dexamethasone, R848, or CpG-ODNs was started in the 4^th^ week of allergen challenge. CpG-ODNs and R848 were given i.tr. (1 mg/kg) and dexamethasone was administered orally (1 m/kg) as shown in Figure 1C. Treatment was given twice weekly one hour prior to allergen challenge. 24 h after the last allergen challenge mice were sacrificed and absolute numbers of total cells, eosinophils, neutrophils and macrophages (A) were measured in whole lung lavage. Data are presented as mean ± SEM, n  =  8–12/group. Lung function was assessed 22 h after final challenge on day 70 via invasive measurement of resistance (B) and compliance (D). The area under the curve (AUC) is shown for resistance (C) and compliance (E). Results represent mean ± SEM for 8–12 mice/group. *P<0.05; **P<0.01; ***P<0.001 in comparison to the PBS control mice and ^§§^ *P*<0.01, ^§§§^ *P*<0.001 in comparison to the triple allergen combination (TAC) group.

**Figure 6:**
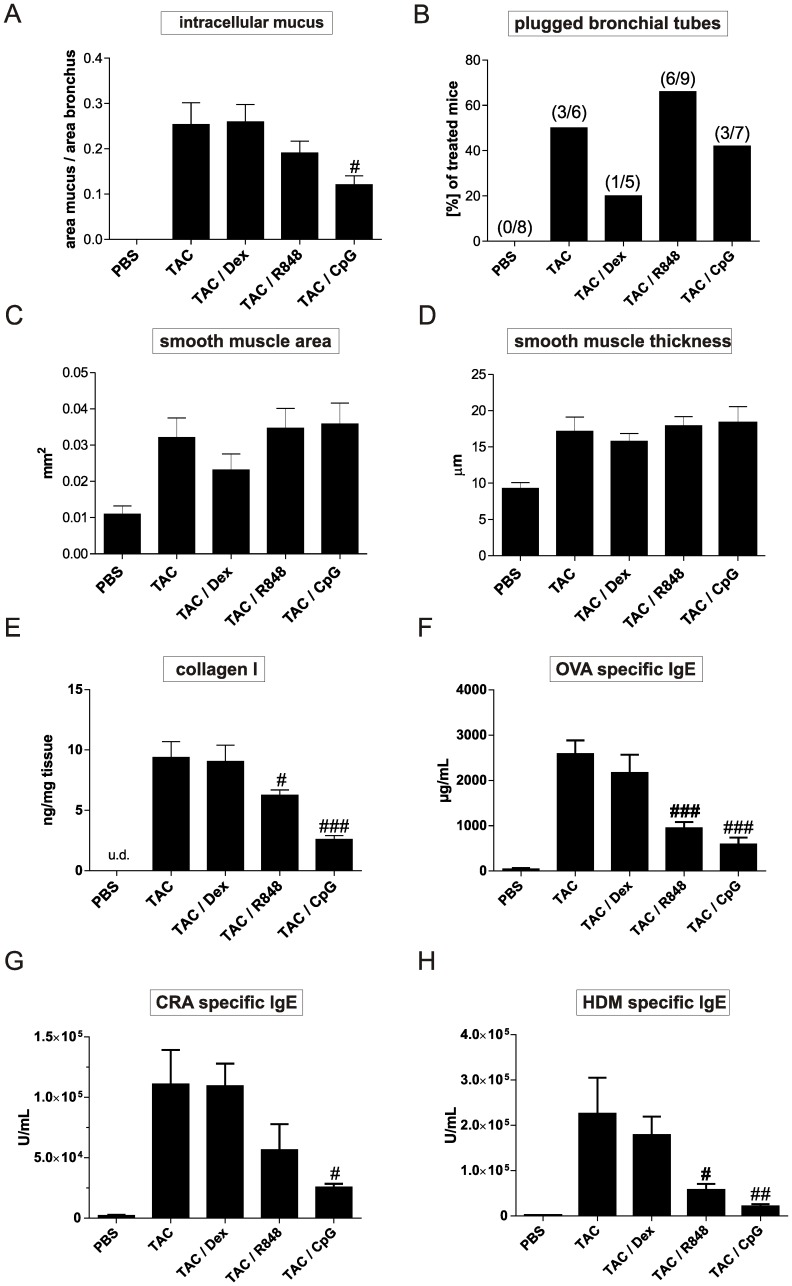
Lung remodeling and allergen specific IgE levels in mice treated with dexamethasone, TLR7 or TLR9 agonists. Mice were treated as described in Figure 1C and in the legend of Figure 5. Mucus production was assessed by automated analysis of the epithelium of PAS stained sections (A). Mucus plugging was scored manually analyzing whole lung cross sections as described in methods (B). Smooth muscle area (C) and smooth muscle thickness (D) were assessed by automated analysis of the smooth muscles surrounding the main bronchus in lungs stained for α-smooth muscle actin. Collagen I levels were determined in lung homogenate via ELISA (E). Levels of IgE specific for the allergens OVA (F), CRA (G), and HDM (H) were measured in the serum of animals and are expressed in arbitrary units. Results represent mean ± SEM for 8–12 mice/group and 5–9 mice/group for mucus plugging. #*P*<0.05; ##*P*<0.01; ###*P*<0.001 in comparison to the triple allergen combination (TAC) group.

Dexamethasone and TLR7 and –9 agonists were applied twice weekly, one hour before allergen challenge. Therapeutic application of dexamethasone had no suppressive effects on any of the parameters measured in the combination model (Table 2, Figure 5 and 6). In contrast, the same dexamethasone treatment significantly reduced influx of total cells, eosinophils and macrophages when applied in our chronic OVA model (Figure S2). Analysis of cell influx into the BAL showed that both TLR agonists significantly reduced lung eosinophilia (TLR7 agonist by 55% and TLR9 agonist by 91%). The TLR7 agonist had no effect on macrophage numbers, whereas the treatment with the TLR9 agonist induced a significant increase (Figure 5D). The two TLR agonists did not reduce the number of neutrophils in the BAL (Figure 5C). Furthermore, TLR9 treatment reduced the levels of both, IL-4 and IL-5 and increased the levels of the pro-inflammatory cytokines IFN-γ, IL-1ß and IL-12 in the BAL (Table 2). The TLR7 agonist had no effect on these parameters. Interestingly, IL-10 levels were 10 fold enhanced in the lung of the TLR9 treated mice. OVA, HDM and CRA specific IgE levels were reduced in both TLR agonist treated groups, with TLR9 having the strongest suppressive effect (Figure 6F, G, H). Analysis of resistance and compliance showed that none of the treatments significantly reduced the loss of airway function (Figure 5 B-E). Histological sections (Figure S1) and measurement of intracellular mucus and smooth muscle mass (Figure 6) showed that smooth muscle thickening was not reduced by any of the treatments. Only the application of the TLR9 agonist reduced the amount of mucus (Figure 6A, Figure S1B) and collagen I in the lung (Figure 6E).

**Table 2:** IL-4, IL-5, IL-10, TGF- β 1, IFN-γ, IL-2, IL-12, and IL-1β cytokine levels in the lungs of mice treated with dexamethasone, TLR7 or TLR9 agonists in the triple allergen challenge model (TAC).

	TAC	TAC/Dex	TAC/R848	TAC/CpG
**IL-4** [pg/ml]	402.7±48.14	369.4±77.26	390.4±70.62	84.42±16.76 (*)
**IL-5** [pg/ml]	687.7±210.3	409.9±79.52	540.4±81.16	177.9±54.19 (*)
**IL-10** [pg/ml]	221.0±119.9	226.8±141.4	428.2±125.0	2355±400.3 (***)
**TGF-ß1** [ng/ml]	196.8±45.26	430.2±171.2	370.3±89.30	198.8±45.40
**IFN-γ** [pg/ml]	71.71±21.47	51.56±13.37	116.1±11.04	210.9±65.80 (*)
**IL-2** [pg/ml]	122.7±37.34	90.68±36.76	228.7±26.38	184.1±41.75
**IL-12** [pg/ml]	6.59±1.52	5.79±2.16	14.48±1.723	59.72±11.64 (***)
**IL-1ß** [pg/ml]	3.21±0.77	2.67±0.52	3.43±0.37	10.37±2.23 (*)

Treatment was started in the 4^th^ week of allergen challenge with 1 mg/kg dexamethasone given orally, 1 mg/kg of R848, or 1 mg/kg CpG-ODNs, both given intratracheal. Values are expressed as mean ± SEM from 8–12 mice/group. *P <0.05, ***P<0.001 in comparison to the triple allergen combination (TAC) group.

## Discussion

The aim of our study was to identify an immunization protocol which causes aspects of a severe asthma phenotype in mice in order to be able to test new therapeutics within a reasonable time frame and with a sufficiently robust assay window. We directly compared the use of OVA, HDM and CRA which, for sensitization, were applied intraperitoneally together with alum. One of the reasons we chose this strategy versus a lung allergen only challenge model was that we have compared the remodeling phenotype in mice between OVA/alum + OVA lung challenge versus OVA lung challenge only. We found that the intraperitoneal OVA/alum sensitization leads to a much stronger remodeling phenotype in a shorter period of time in comparison to a mucosal sensitization (data not shown). A recent publication supports our finding that intraperitoneal allergen sensitization together with alum induces a much stronger allergic inflammation in the lung compared to lung challenge only [19]. We are aware that mucosal sensitization only has been shown to induce allergic reactions in the lung of mice and it was argued that this mimics the clinical situation more closely than using allergen + adjuvant [22]. However, in our opinion mouse models generally cannot perfectly mimic the complex human asthma situation. For this reason we chose to focus on the phenotype generated in the lungs and not how it was generated. A second reason we chose to use the combination allergen + alum was that we hoped to be able to generate a phenotype which is at least to a certain degree steroid resistant because we hypothesized that only a very strong severe asthmatic phenotype in mice may be resistant to dexamethasone treatment. We found that all three allergens applied alone induced strong Th2-type airway inflammation, airway hyperreactivity and airway remodeling with no major difference between OVA and CRA, and a slightly stronger phenotype with HDM in some parameters. These included fibrosis in particular. In agreement with a previously published report [20], we also found that the OVA and HDM model induced comparable levels of IL-4 and IL-5 in the lung.

When mice were immunized with all three allergens simultaneously and then challenged with allergen (alternating between the three allergens) a stronger remodeling, albeit not significant phenotype, greater Th2 response and increased airway hyperreactivity compared to only using the single allergens was observed. Our experiments were not designed to address the question of whether triple allergen combinations are generally better at inducing Th2 responses than when using a single allergen. This clearly depends on the model used, the degree of endotoxin contamination in the allergen preparations, the amounts of allergen applied and other factors. However, using our protocol, triple allergen combination is clearly superior to the use of single allergens alone when analysing all parameters. A recent report has also shown that combining allergens in one asthma model can induce severe disease and this was associated with stopping the development of allergen-induced tolerance [21]. Using more than one allergen may also reflect the human situation better where most asthmatics are allergic to more than one allergen.

A surprising finding was that although the triple combination induced the strongest Th2 response in the lung with the highest IL-13 levels detected (known to directly induce mucus production and goblet cell metaplasia) we did not observe the highest goblet cell hyperplasia and amounts of intracellular mucus in the main bronchus. However, mice in the combination model showed the most severe mucus plugging. The lower amount of mucus staining in the combination group may be explained by an increase in mucus secretion into the airways leading to the observed increased plugging.

Mice treated with all three allergens also showed the highest airway hyperreactivity and airway constriction at baseline. This is most likely due to the mucus plugging and enhanced levels of IL-13, increased smooth muscle mass and increase in fibrosis, as all these factors have been shown to contribute to AHR [22].

Overexpression of TGF-β1 in mice was shown to cause lung fibrosis [23]. The correlation between pronounced fibrosis and TGF-β1 levels in the lung of the HDM and combination treated mice suggest that TGF-β1 may contribute to the fibrosis in this model.

Why does the combination of three allergens lead to a stronger Th2 response than when using only one allergen? One reason may be that an increased amount of epitopes for Th2 cell activation is present inducing the stronger Th2 responses observed. The strong increase in IL-2 in the BAL supports the view of stronger T cell activation. IL-2 may also be directly responsible for the enhanced Th2 response as shown previously [24], [25]. Although enhanced Th1 responses are usually associated with a reduction in Th2 responses the detected increase in IFN-γ and IL-12 in the combination model suggests an increased Th1 response which may also be contributing to the increased pathology. This is supported by clinical studies that show that increased IFN-γ levels in lavage of asthmatics correlate with severity of disease [26]–[29], and that IFN-γ can increase the production of IL-5, IL-13 and airway eosinophilia [30]. In the combination model both, a strong Th2 and a significant Th1 response co-developed.

A major problem for patients suffering from severe asthma is the reduced efficacy of first line steroid treatment, leading to higher dosing or insensitivity to treatment. However, at present there are no allergic asthma mouse models reported reflecting these clinical findings. Published studies using OVA [31], CRA [32], [33], and HDM [34] in either acute or chronic setting all report significant reduction of Th2 responses by dexamethasone treatment. We applied a high dose of the steroid dexamethasone as well as TLR7 and TLR9 agonists in the combination model to investigate if they can reduce the observed inflammation and pathology. We used a dexamethasone treatment protocol that reduced OVA-induced eosinophilia and IL-4 production in the lung by greater than 95% when given 1 h prior to allergen challenge in an acute 4 week asthma model (3 x OVA + alum intraperitoneal followed by 2 airway challenges, data not shown) and also strongly reduced the lung inflammation when OVA was used as allergen in a chronic model (Figure S2). In the combination model, however, we found that dexamethasone had no significant suppressive effect on any of the parameters measured. This was very surprising, and we can only speculate as to why dexamethasone had no suppressive effects. It is possible that the increased levels of IL-13 may be contributing to the steroid insensitivity as reported in a model with adenoviral induced IL-13 over expression [35]. Furthermore, the observed increased IFN-γ may be synergizing with IL-27, resulting in steroid insensitivity as suggested previously [36]. It has also been shown that Th17 cells may also contribute to steroid insensitivity in asthma [37]. A small significant increase in IL-17 and neutrophil numbers (possibly associated with IL-17) was also detected in the combination model. However, in the OVA model, which is steroid sensitive, higher IL-17 levels were detected, suggesting that IL-17 alone cannot be responsible for the steroid resistance observed. Further studies are needed to address the question if IL-13, IL-17, IFN-γ/IL-27 or other factors are responsible for the steroid insensitive phenotype. Using a model published by Wilson et al., demonstrating a strong Th17 response after inhalative challenge only may be more suited to address the question if IL-17 leads to steroid insensitivity [38]

Although, the amount of dexamethasone applied reduced the OVA-induced Th2 inflammation in a chronic setting but not when using the triple allergen model, we cannot conclude that is due to using all three allergens. When we used HDM or CRA a stronger inflammation and remodeling phenotype was also induced compared to when using OVA. It is possible that models using CRA and or HDM may also be dexamethasone resistant.

In contrast to the lack of suppressive effects of dexamethasone we found that both the TLR7- and TLR9 agonist were able to reduce some features of the asthmatic phenotype. The TLR7 agonist reduced the influx of eosinophils and allergen-specific IgE levels in the serum. The TLR9 agonist had stronger effects on these parameters and in addition reduced mucus production in the lung and IL-4 and IL-5 levels in the BAL. Neither of the TLR agonists was able to reduce the AHR or the increase in smooth muscle mass. The TLR9 agonist treated mice showed an increase in lung neutrophilia, IFN-γ, IL-1ß and IL-12 levels in the homogenate, indicative of increased Th1/innate responses, suggesting that the TLR9 mediated suppressive effects on the Th2 response may be due to enhanced Th1 or innate responses as discussed previously [13]. In contrast, increased Th1 responses in the combination model correlated with disease severity and not protection. Therefore the type or quality of the Th1 response induced by the allergens in contrast to the TLR9 agonist may be different. Another explanation may be that the observed tenfold induction of IL-10 by the TLR9 agonist in the lung is responsible for the reduced Th2 phenotype. Supporting this finding is that the reduction of allergic inflammation by the helminth *Nippostrongylus brasiliensis* was mediated by IL-10 [39]. Nevertheless, in light of our own published data [13] together with numerous other publications in rodents and monkeys showing strong anti-allergy effects of these two agonists [12], [40]–[45], the observed moderate suppression in the combination model was surprising and attests to the difficult to treat phenotype that was induced.

Taken together we show that using a triple allergen combination with OVA, CRA and HDM for sensitization and allergen challenge, leads to a very severe human asthma like phenotype in the lungs of mice stronger than when using the allergens alone. The phenotype induced reflects the atopic phenotype with a strong Th2 plus weaker Th1 response. This chronic model is steroid insensitive and less sensitive to therapeutic intervention with the TLR7 and TLR9 agonist resiquimod or CpG-ODN than other published severe asthma models [12], [40], [46], [47]. Nevertheless, the strongest suppressive effects were found when the TLR9 agonist was used suggesting that TLR9 agonists may also be effective in treating steroid insensitive severe asthma patients. Our novel triple allergen combination model may be useful to test new drug candidates aiming to treat steroid insensitive atopic severe asthma in humans and to investigate the mechanisms responsible for the development of reduced steroid sensitivity.

## Supporting Information

Figure S1 **Histological sections of the lung of mice treated with dexamethasone, TLR7 or TLR9 agonists in triple allergen treated mice (TAC).** Representative sections of haematoxilin/eosin (H&E) (A), periodic acid-Schiff (PAS) (B), and sirius red staining of the main bronchus with surrounding area (C) are shown. Scale bar  =  100 µm. Shown are representative sections of 8-12 mice per group. Triple allergen combination (TAC) group.
(TIF)

Figure S2 **Dexamethasone reduces OVA induced cellular influx in a chronic model.** Mice were treated with OVA as described in Figure 1A and with dexamethasone as described in the legend of Figure 5. 24 h after the last allergen challenge mice were sacrificed and absolute numbers of total cells, eosinophils, and macrophages in the OVA model were measured in whole lung lavage. Data are presented as mean ± SEM, n  =  8-12/group. Results represent mean ± SEM for 8-12 mice/group. *P<0.05; **P<0.01; ***P<0.001 in comparison to the PBS control mice and ^§^ *P*<0.05; ^§§^ *P*<0.01, ^§§§^ *P*<0.001 in comparison to the OVA group.
(TIF)
