# Supplementary figures and images for: Development of a Novel Severe Triple Allergen Asthma Model in Mice Which Is Resistant to Dexamethasone and Partially Resistant to TLR7 and TLR9 Agonist Treatment
Source: PLoS One. 2014 Mar 11;9(3):e91223. doi: 10.1371/journal.pone.0091223 (PMC3949744; doi:10.1371/journal.pone.0091223)

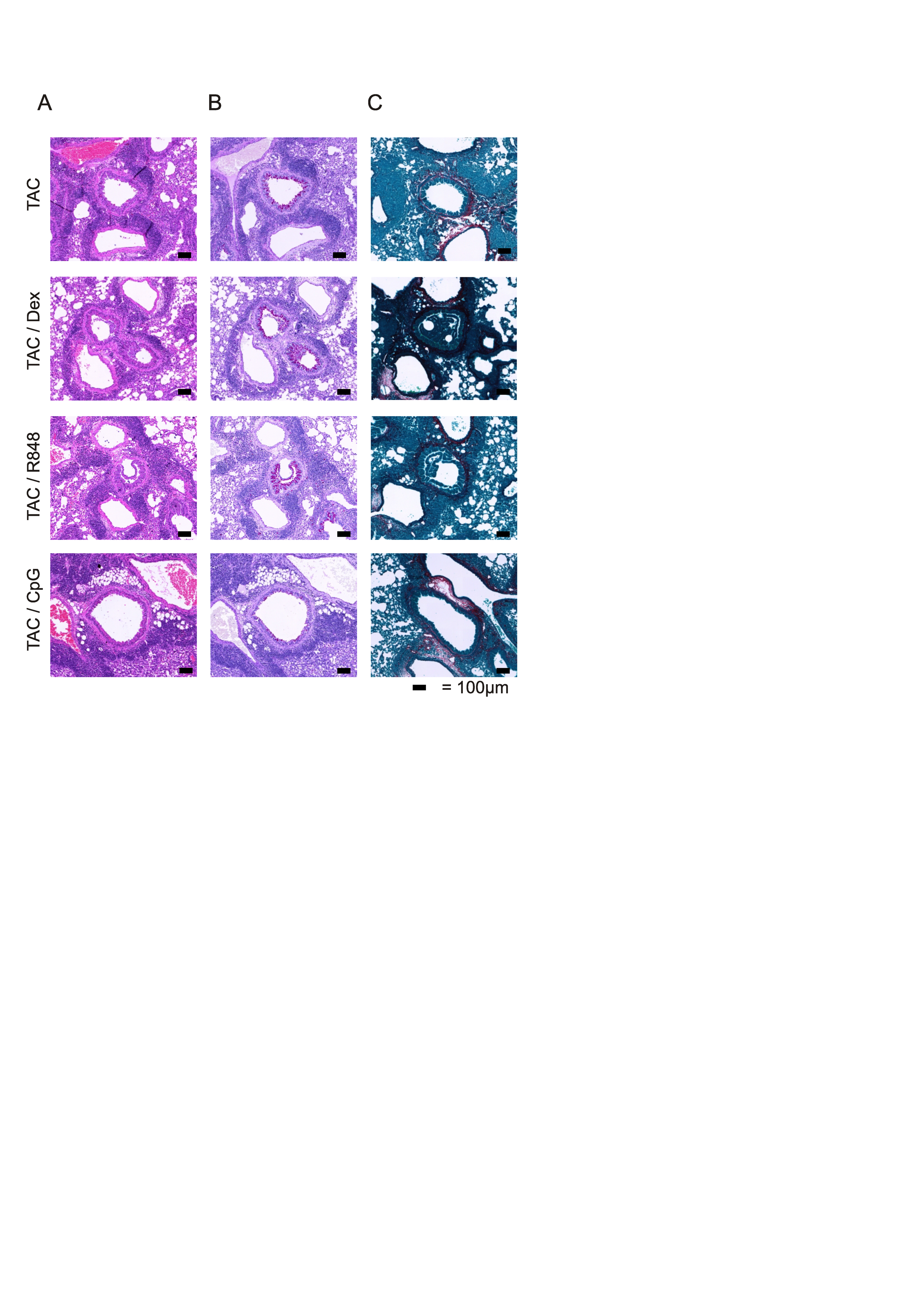

Supplement: Figure S1 — Histological sections of the lung of mice treated with dexamethasone, TLR7 or TLR9 agonists in triple allergen treated mice (TAC). Representative sections of haematoxilin/eosin (H&E) (A), periodic acid-Schiff (PAS) (B), and sirius red staining of the main bronchus with surrounding area (C) are shown. Scale bar = 100 µm. Shown are representative sections of 8-12 mice per group. Triple allergen combination (TAC) group. (TIF) [file pone.0091223.s001.tif]

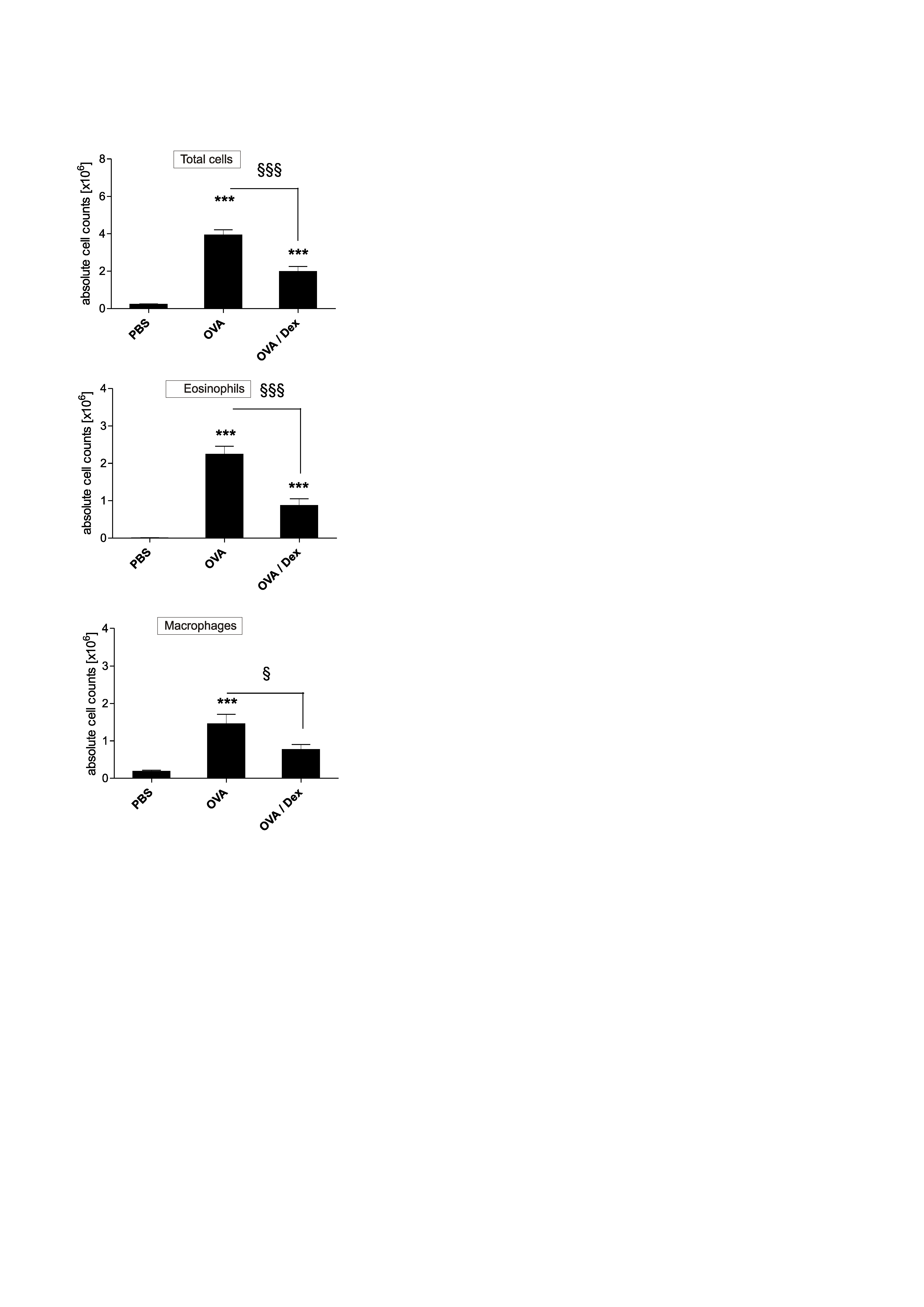

Supplement: Figure S2 — Dexamethasone reduces OVA induced cellular influx in a chronic model. Mice were treated with OVA as described in Figure 1A and with dexamethasone as described in the legend of Figure 5. 24 h after the last allergen challenge mice were sacrificed and absolute numbers of total cells, eosinophils, and macrophages in the OVA model were measured in whole lung lavage. Data are presented as mean ± SEM, n = 8-12/group. Results represent mean ± SEM for 8-12 mice/group. *P<0.05; **P<0.01; ***P<0.001 in comparison to the PBS control mice and § P<0.05; §§ P<0.01, §§§ P<0.001 in comparison to the OVA group. (TIF) [file pone.0091223.s002.tif]
